# Laboratory development of an RNA quantitative RT-PCR assay reporting in international units for hepatitis D virus

**DOI:** 10.3389/fmicb.2024.1472826

**Published:** 2024-11-20

**Authors:** Carla Osiowy, Jacqueline Day, Emma R. Lee

**Affiliations:** ^1^National Microbiology Laboratory, Public Health Agency of Canada, Winnipeg, MB, Canada; ^2^Department of Medical Microbiology and Infectious Diseases, University of Manitoba, Winnipeg, MB, Canada

**Keywords:** hepatitis D virus, RNA, viral load, IU/mL, validation, diagnostic

## Abstract

**Introduction:**

Chronic hepatitis D virus (HDV) is associated with rapid progression to severe liver disease. Co-infection with HDV and hepatitis B virus is likely underdiagnosed due to challenges in diagnostic test availability and standardization. With new HDV antiviral options, HDV RNA quantification is essential for understanding the patient response to treatment. To this end, a quantitative real-time reverse transcription PCR (qRT-PCR) assay utilizing synthetic RNA calibrators and a conversion factor to quantify HDV RNA in WHO international standard units (IU/mL) was developed and validated.

**Methods:**

qRT-PCR primers and probes were selected within the ribozyme region. Thermocycling conditions and reactions were optimized. Synthetic RNA transcripts were prepared as quantification standards and calibrators. Transcript dilutions (log_10_ 8 to log_10_ 1 copies/μL) were calibrated against the WHO standard and a conversion factor calculated to convert copies/μL to IU/mL. Assay validation and evaluation was conducted, including use of specimens from 8 HDV genotypes and comparison to a commercial assay.

**Results:**

The assay lower limit of detection was determined by probit analysis to be 11 IU/mL (8.63–15.78 95% CI). Inter- and intra-assay coefficient of variation analysis showed 96.6% precision and 90.6% accuracy. A conversion factor of 16.5 was used to convert copies/μL to IU/mL. All 8 HDV genotypes were quantified by the assay and commercial assay comparison showed good agreement.

**Discussion:**

The developed assay has clinical utility for the sensitive and specific quantitative monitoring of HDV RNA, appropriate for medium to high throughput laboratories.

## 1 Introduction

The estimated prevalence of hepatitis D virus (HDV) seropositivity among hepatitis B surface antigen (HBsAg) positive Canadians is thought to be approximately 3 to 5% based on modeling (Polaris Observatory Collaborators, 2024), weighted measurements via meta-analyses (Wong et al., 2024) and referred patient testing (Osiowy et al., 2022). HDV global prevalence has been estimated to range from 4.5% to approximately 13% (Miao et al., 2020; Stockdale et al., 2020; Chen et al., 2019); however, a recent study described lower rates among 25 countries and territories in comparison to previous estimates, except for Canada and France, in which prevalence rates were higher (Polaris Observatory Collaborators, 2024). Certain countries and regions are known to have endemic or hyper-endemic levels (>10%) of HDV infection, such as parts of Eastern Europe, Central Asia, Central Africa, and the Amazon Basin (Lee and Lee, 2021; Rizzetto et al., 2021). Canada is among several countries in which the epidemiology of HDV is shifting such that rates of infection remain stable or are increasing due to immigration from regions of HDV endemicity (Caviglia et al., 2022; Tian et al., 2022).

Chronic hepatitis D infection (CHD) is the most aggressive form of viral hepatitis resulting in an increased risk of developing hepatocellular carcinoma compared to those mono-infected with hepatitis B virus (Wranke et al., 2024) with a pooled odds ratio of 2.77 (95% CI 1.79–4.28) among prospective studies (Alfaiate et al., 2020). As such, it is essential that standardized screening of HBsAg-positive persons is implemented to identify CHD patients for proper linkage to care and management. Although PEG-IFN alpha is the only treatment currently approved for CHD in Canada (Coffin et al., 2018), several antiviral therapies, such as bulevirtide and lonafarnib, are in Phase 2 or 3 clinical trials (Yurdaydin et al., 2022; Asselah et al., 2024) or have been conditionally approved by regional authorities (Dietz-Fricke et al., 2023) for treatment of CHD. As these new therapies become available, it is imperative that standard assays for HDV RNA viral load measurement having robust performance characteristics are used for monitoring treatment response and management of CHD.

This study describes the development and validation of a sensitive one-step quantitative real-time reverse transcription PCR (qRT-PCR) method for measurement of HDV RNA in international units per mL (IU/mL). Quantification using the World Health Organization (WHO) RNA standard allows comparison of RNA levels across different studies and laboratories (Wedemeyer et al., 2023). The described method demonstrated excellent sensitivity for all 8 HDV genotypes, with robustness and ease of use suitable for a medium to high-throughput laboratory.

## 2 Materials and methods

### 2.1 Study specimens

The 1^st^ World Health Organization (WHO) International Standard for HDV RNA amplification (Paul Ehrlich Institut code 7657/12, Langen, Germany) was reconstituted using sterile nuclease-free water to a concentration of 5.75E+05 IU/mL as described in the reference material insert (Paul-Ehrlich-Institut Regulation WHO Reference Material, 2024) and used for assay validation purposes. Select clinical specimens submitted to the National Microbiology Laboratory for HDV RNA reference diagnostic testing were used to develop and validate the HDV viral load assay. Approximately 2 to 6 clinical specimens of each HDV genotype (gt 1, 2, 5–8) and 2 synthetic double stranded DNA fragments (gBlock Gene Fragments; Integrated DNA Technologies, Ottawa, ON) for HDV genotypes 3 and 4 were employed for specificity analysis. gBlock fragments were designed using HDV gt 3 sequence accession no. HF679405 (nt 769–957) and HDV gt4 sequence accession no. AB118847 (nt 766–963). Clinical specimens were genotyped by phylogenetic analysis, as described previously (Osiowy et al., 2022). Subgenotyping of specimens was not performed.

### 2.2 Nucleic acid extraction

HDV RNA was extracted from 200 μL clinical specimen (serum or plasma) or reconstituted HDV standard using an automated nucleic acid extraction system (NucliSENS easyMag, bioMerieux Inc., Saint-Laurent, QC) and eluted in 55 μL elution buffer. AcroMetrix EDTA plasma dilution matrix (ThermoFisher Scientific, Mississauga, ON) was used as a negative extraction control for each set of clinical specimen extractions.

### 2.3 HDV RNA quantitative calibration standard preparation

RNA standard material was prepared by transcription from a target site HDV amplicon using T7 polymerase (TranscriptAid T7 high yield kit; ThermoFisher Scientific) according to the manufacturer’s instructions. The target site amplicon (approximately 650 bp) was prepared by RT-PCR (Superscript IV OneStep RT-PCR enzyme mix; ThermoFisher Scientific) using the primers listed in Table 1 for RNA transcript synthesis and target RNA extracted from a genotype 1 clinical serum sample (GenBank accession no. PP943060). Following T7 polymerase transcription, the reaction mixture was treated with 1 μL DNase (Baseline-ZERO, 1 MBU/μL; Lucigen, Middleton, WI) to remove amplicon DNA and the RNA purified using paramagnetic bead-based chemistry (RNAClean XP beads; Beckman Coulter, Indianapolis, IN) and eluted in RNA Storage solution (ThermoFisher Scientific). RNA quality and concentration was determined using RNA ScreenTape (TapeStation 4,200; Agilent, Santa Clara, CA) and a Qubit fluorometer (ThermoFisher Scientific), respectively. RNA copies/μL were calculated using the ng/μL concentration and an online calculator (New England Biolabs).^1^ Synthetic HDV RNA was diluted 10-fold in RNA Storage solution and aliquoted to obtain working standards ranging from 8.38 log_10_ to 1.38 log_10_ copies/μL. All RNA was stored at −80°C.

**Table 1:** List of primers and probe used in the study.

Primer	Sequence (5′–3′)	nt range (D01075^1^)	Purpose^2^
DeltaF2	CTCCCTTWGCCATCCGAG	819-836	HDV RNA quantification Fwd primer
DeltaRnew	TCTTCGGGTCGGCATG	892–907	HDV RNA quantification Rev. primer
DeltaProbe2	CGGATGCCCAGGTCGGAC	855–872	HDV RNA quantification Probe
T7HDV678f	TAATACGACTCACTATAGGGAGAGATGGCCGGCATGGTCCCAG	685–704	RNA transcript synthesis
HDV1326r	CTCAGGGGAGGATTCACCGACA	1,312–1,333	RNA transcript synthesis

1 GenBank accession number.

2 Fwd, forward; Rev, reverse.

### 2.4 Quantification of HDV RNA

Extracted HDV RNA or gBlock fragments were quantified by the one-step real-time RT-PCR method using primers and probe targeting the ribozyme coding region (nucleotide 819–907, according to GenBank accession number D01075), described in Table 1. Primers DeltaRnew and DeltaProbe2 are slight modifications of the ‘Delta-R’ and ‘Delta-P’ primers described by Le Gal et al. (2005) to improve primer melting temperatures and hybridization for all HDV genotypes. The ribozyme coding region is the most highly conserved HDV genomic region among all HDV genotypes (Huang and Lo, 2010), and thus is the most practical selection for primer hybridization. One-step qRT-PCR was performed using TaqPath qPCR 4X Master Mix (ThermoFisher Scientific), with primers and probe at a final concentration of 0.9 μM and 0.2 μM, respectively, in a reaction volume of 20 μL including 14 μL extracted RNA. Extracted EDTA plasma dilution matrix and nuclease-free water were included as negative controls in each amplification run. Thermocycling proceeded using an Applied Biosystems QuantStudio thermocycler (ThermoFisher Scientific) selected for FAM detection with an initial UNG inactivation (2 min at 25°C) followed by reverse transcription (15 min at 50°C), enzyme activation (2 min at 95°C), and 40 cycles of amplification (each cycle consisting of 15 s at 95°C and 45 s at 60°C).

Initial development of the qRT-PCR assay involved running a standard curve of synthetic RNA dilutions (8.38 log_10_ to 1.38 log_10_ copies/μL) in triplicate alongside all clinical RNA extracts as well as duplicate measurements of the extracted WHO HDV RNA standard at 1.76, 2.76 and 4.76 log_10_ IU/mL. The synthetic RNA standards were used to initially quantify HDV RNA as copies/μL. This also allowed a calculation of the appropriate conversion factor from copies/μL to IU/mL. Once a conversion factor was established, the final developed assay utilized two synthetic RNA calibrators (2.38 log_10_ copies/μL and 7.38 log_10_ copies/μL) and the extracted WHO HDV RNA standard diluted to 1.76, 2.76, and 4.76 log_10_ IU/mL, each measured in duplicate with each run. The copies/μL computed following thermocycling was multiplied by the calculated conversion factor 16.5 (see below) to obtain the RNA quantity in IU/mL.

A commercial HDV RNA quantification assay (Eurobioplex HDV qRT-PCR, Eurobio Scientific, Les Ulis, France) was run on a panel of 15 HDV seropositive specimens according to the kit instructions in parallel with the one-step assay for evaluation purposes.

### 2.5 Quality control of HDV RNA measurements

Historical values of all calibrators (Ct for synthetic RNA and calculated IU/mL for WHO HDV RNA standards) were used to calculate the weighted average and total SD (square root of total variance) of each calibrator following one-way ANOVA analysis of approximately 25 observations grouped by year for 3 years and performed by 3 different operators. Thereafter, calibrator values were tracked per run using Levey-Jennings control charts of the weighted average. Run values were required to fit within one standard deviation of the mean calculated log_10_ IU/mL and 2 standard deviations of the copies/μL mean Ct for the run to pass, otherwise the run would be repeated.

### 2.6 Calculation of the conversion factor from copies/μL to IU/mL

The mean IU/mL was calculated for each synthetic HDV RNA standard dilution (8.38 log_10_ to 1.38 log_10_ copies/μL) using historical data from approximately 20 separate runs over a 3-year period involving 3 different operators using the one-step qRT-PCR method. IU/mL was calculated from the slope and intercept of the line created by plotting the mean Ct from replicate runs of the WHO HDV RNA standards 1.76, 2.76 and 4.76 log_10_ IU/mL run alongside synthetic HDV RNA standard dilutions over the same period of time. The calculated IU/mL for each standard dilution was divided by the copies/μL of each standard. The conversion factor was determined by averaging the quotient of each standard.

### 2.7 Limit of detection and quantification

The analytical sensitivity of the assay was determined by preparing serial 10-fold dilutions of the WHO HDV RNA standard from 2.76 log_10_ IU/mL to −1.24 log_10_ IU/mL and from 2.06 log_10_ IU/mL to −0.94 log_10_ IU/mL. Dilutions of reconstituted standard were performed in negative serum matrix (AcroMetrix EDTA plasma dilution matrix; ThermoFisher Scientific). Eight replicates of the dilution series (9 dilutions in total per series) were prepared, and each dilution was extracted and run in triplicate using the one-step qRT-PCR method to result in 24 replicates for probit analysis.

### 2.8 Linearity

A clinical specimen having high viral load calculated in copies/mL was diluted 10-fold (7.62 log_10_ to 1.62 log_10_), extracted, and the RNA ran in quadruplicate on two separate days. The log_10_ copies/mL was plotted against the log_10_ IU/mL of each replicate dilution, calculated based on the slope and intercept resulting from extracted WHO HDV RNA standard Ct values.

### 2.9 Inter-assay precision

A clinical specimen having high viral load calculated in IU/mL was diluted to 5.5 log_10_ (‘High’) and 2.5 log_10_ (‘Low’) IU/mL. Each dilution was run in duplicate for 8 days.

### 2.10 Intra-assay variation

A clinical specimen pool (all genotype 1) having viral load calculated in IU/mL was diluted to create 10 replicates each of 5.1 log_10_ (‘High’) and 1.1 log_10_ (‘Low’) IU/mL. Each set of replicates for each dilution was run at one time and the %CV determined.

### 2.11 Statistical analysis

Statistical analysis of performance characteristic parameters was performed using GraphPad Prism v.10.1.1. Analysis of results for the limit of detection and quantification was performed by Probit, and nonlinear regression model analysis [asymmetric sigmoidal, 5PL X is log(concentration) equation] was used to interpolate the lower limit of detection (LLoD) from the 95% probability value (Clinical and Laboratory Standards Institute, 2017). The mean Ct values of the 9 WHO HDV RNA standard dilutions were plotted to determine the lower limit of quantification (LLoQ) from the linear portion of the curve. Analysis of results for linearity was performed by linear regression. Inter-assay precision analysis involved calculating the percent coefficient of variation (%CV) for the mean log_10_ value for the High and Low specimen dilutions each day for each dilution run. Intra-assay variation analysis was performed by determining the % CV of High and Low pooled specimen replicates run at one time and the 95% confidence interval of the mean IU/mL was plotted for each group of replicates.

### 2.12 Ethical approval

The testing of patient specimens enrolled in the Canadian Hepatitis B Network was approved by the Health Canada and Public Health Agency of Canada REB (protocol ID REB 2019-036P) and the University of Manitoba institutional ethics review board (protocol ID H2020:403). No patient identifier information was involved in this study.

## 3 Results

### 3.1 Performance characteristics of the one-step qRT-PCR HDV quantification assay

**Figure 1:**
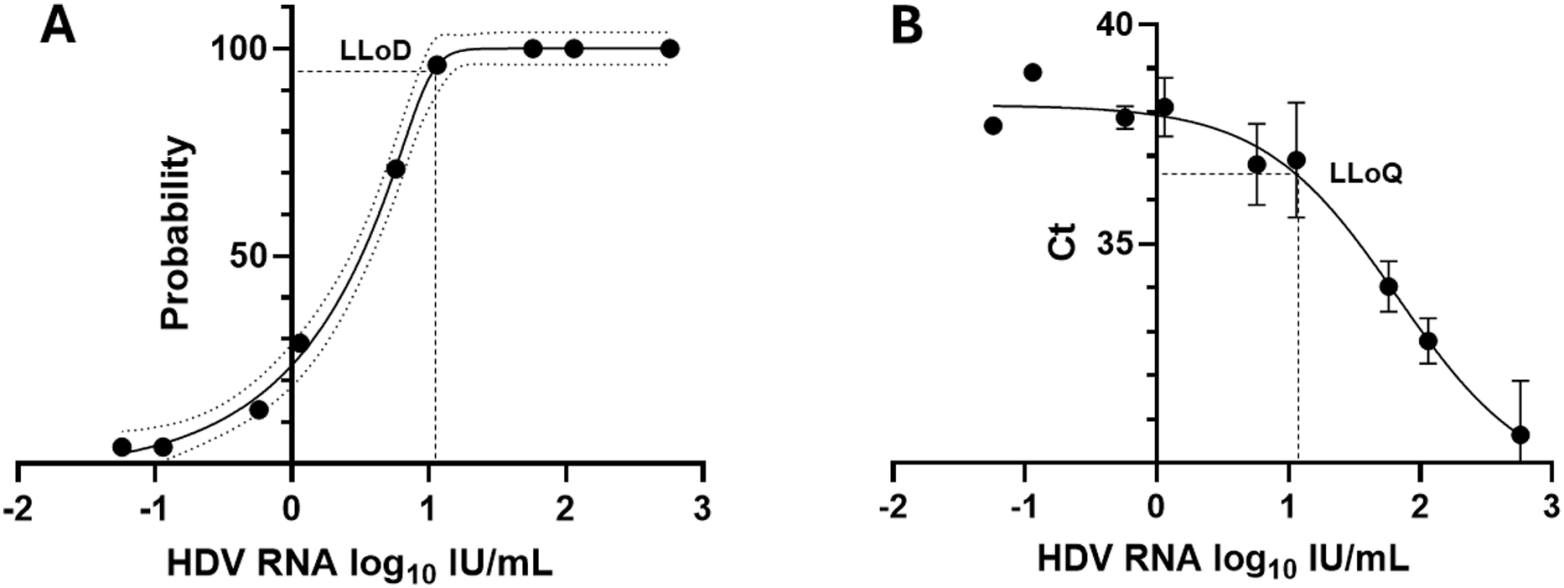
Lower limits of detection and quantification for the HDV RNA one-step qRT-PCR method. (A) Twenty-four replicates resulting from 9 concentrations of the WHO HDV RNA Standard were measured using the one-step qRT-PCR assay. Probit and nonlinear regression analysis were used to interpolate the lower limit of detection (LLoD) from the 95% probability value. Black dots denote the probability of detection (‘hit rate’) for each HDV RNA standard concentration. Dotted lines denote the 95% CI of the data. (B) The Ct mean and standard deviation of each of the nine WHO HDV RNA standards, from 24 replicates, was plotted. The lower limit of quantification (LLoQ; shown as dashed line) was inferred from the onset of the linear portion of the graph. Graphs prepared using GraphPad Prism v.10.1.1.

**Figure 2:**
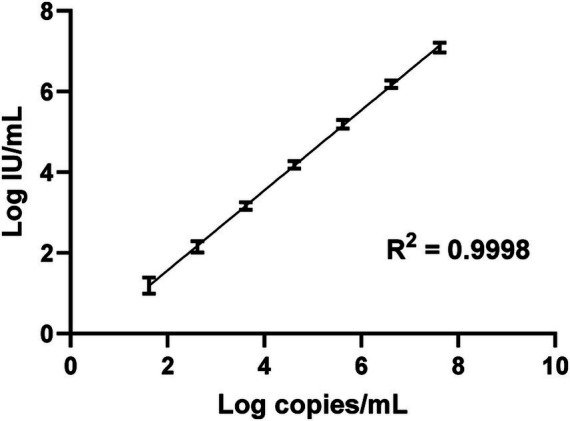
Linearity analysis of the HDV RNA one-step qRT-PCR method. Log_10_ copies/mL of dilutions of a clinical sample were plotted against the log_10_ IU/mL of each replicate dilution, calculated based on the slope and intercept resulting from extracted WHO HDV RNA standard Ct values. Values were analyzed by linear regression and the graph prepared using GraphPad Prism v.10.1.1.

The LLoD of the one-step qRT-PCR assay was 1.041 log_10_ IU/mL (11 IU/mL) with a 95% confidence interval [CI] of 0.936 to 1.198 log_10_ IU/mL (8.63 to 15.78 IU/mL) as shown in Figure 1A. The LLoQ was found to be approximately equivalent to the LLoD (11 IU/mL; Figure 1B) as the end of linearity of the standard curve fell within the 1.06 log_10_ IU/mL point on the curve. Figure 2 shows the linearity and comparison between calculated IU/mL of dilutions of a clinical sample having a high copies/mL viral load, with an R^2^ of 0.9998.

**Figure 3:**
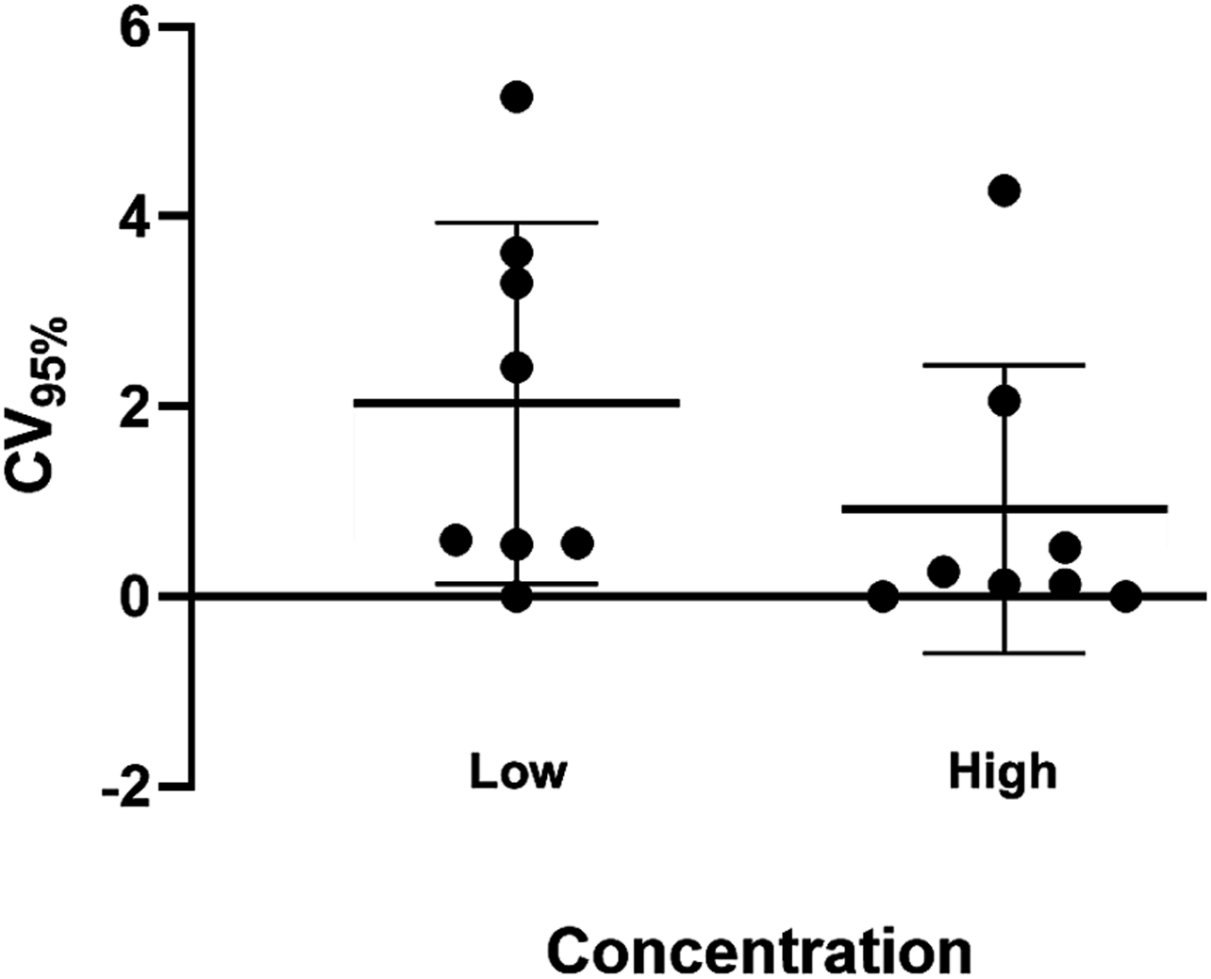
Inter-assay precision of the HDV RNA one-step qRT-PCR method. Two dilutions (Low: 2.5 log_10_ IU/mL; High: 5.5 log_10_ IU/mL) of a quantified clinical sample were run in duplicate for 8 days. The percent coefficient of variation (%CV) for the mean log_10_ value was calculated each day for each dilution. The heavy black bars show the mean CV (95%) for each concentration (Low, 2.04; High, 0.92); the light black bars show the standard deviation of the mean (Low, 1.90; High, 1.52).

**Figure 4:**
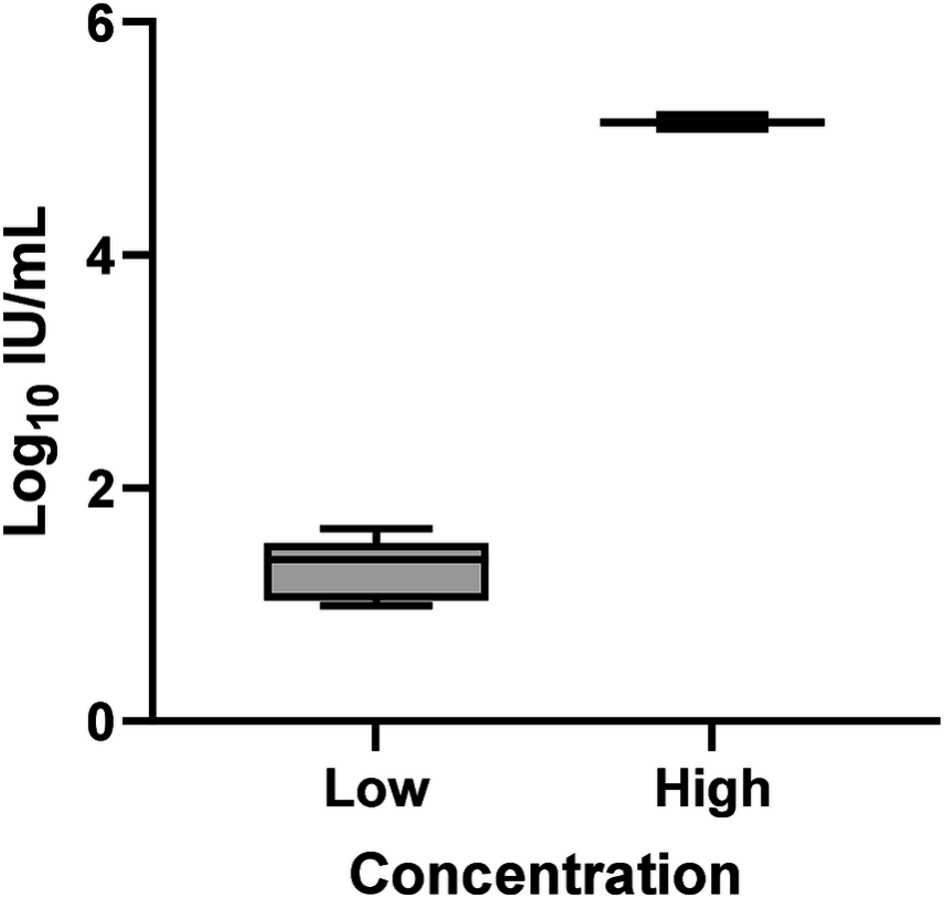
Intra-assay precision of the HDV RNA one-step qRT-PCR method. Ten replicates each of two dilutions (Low: 1.1 log_10_ IU/mL; High: 5.1 log_10_ IU/mL) of a quantified clinical sample were run and the %CV determined. The mean log_10_ IU/mL and 95% confidence interval was plotted for each group of replicates for each concentration. The mean log_10_ IU/mL and standard deviation of the two dilutions following precision analysis was 1.327 ± 0.239 (Low) and 5.141 ± 0.035 (High).

Overall inter-assay precision using two HDV RNA concentrations (‘Low’ 2.5 log_10_ and ‘High’ 5.5 log_10_ IU/mL) was 96.6%, with CV% of both concentrations consistently below 10% (Figure 3). The overall reproducibility within an assay run was 90.6%, with variation more pronounced at lower concentrations (CV% of 18% at ‘Low’ 1.1 log_10_ IU/mL and 0.7% at ‘High’ 5.1 log_10_ IU/mL; Figure 4).

Clinical specimens having known HDV genotype 1, 2, and 5 to 8 (an average of 4 samples per genotype, other than genotype 8; *n* = 1) were available for extraction and qRT-PCR analysis. Ninety-five percent (21/22) of specimens could be detected and quantified by the one-step qRT-PCR method (Supplementary Table 1). As the laboratory did not have a sufficient volume of genotype 3 and 4 specimens available for extraction, linear dsDNA (gBlocks) was synthesized to mimic both genotypes for evaluation of the primers and probe. The material was diluted to 0.01 pg./μL and 0.001 pg./μL and tested in duplicate, with successful detection of both genotypes (Supplementary Table 1).

### 3.2 Determination of conversion factor for copies/μL to IU/mL

The conversion factor from copies/μL to IU/mL was 16.5. This was determined by calculating the mean Ct value for each synthetic RNA standard dilution (8.38 log_10_ to 1.38 log_10_ copies/μL) run over a period of 3 years (approximately 20 determinations per dilution). Using the mean Ct value, the log_10_ IU/mL of each dilution was calculated based on the slope (−3.46) and intercept (40.934) of the line created by plotting the mean Ct from replicate runs of the WHO HDV RNA standards (1.76, 2.76 and 4.76 log_10_ IU/mL) run alongside the synthetic RNA dilutions over time. The calculated log_10_ IU/mL was converted to IU/mL and this value was divided by the copies/μL for the synthetic RNA standard dilutions. The mean quotient of each standard, calculated to be 16.5, was used as the conversion factor (Supplementary Table 2).

### 3.3 Evaluation of the one-step qRT-PCR method in comparison to a commercial method and by an external quality assurance HDV RNA panel

The laboratory developed test (LDT) was evaluated using a commercial qRT-PCR assay reporting in IU/mL (Eurobioplex HDV qRT-PCR) and by participating in an external quality assurance program for HDV RNA detection, in which an IU/mL value is provided for each sample (INSTAND, Düsseldorf, Germany; 2022 EQA #400 HDV RNA panel). A panel of 15 HDV seropositive clinical specimens, including the WHO HDV standard at 2.76 log_10_ IU/mL and genotype 1 and 5 samples, were extracted and RNA quantified by the one-step qRT-PCR method and according to the manufacturer’s instructions for the Eurobioplex assay. Log_10_ IU/mL values were very similar between the two assays, with the one-step assay frequently showing comparatively increased IU/mL for specimens under 2 log_10_ IU/mL according to Eurobioplex quantification, including measurement of a specimen undetectable by the Eurobioplex (Table 2). Several samples showed discrepancy, with one genotype 5 sample (panel sample #8) having greatly reduced quantity by Eurobioplex testing compared to the one-step assay, while another sample (panel sample #6) quantified by the one-step assay was not detected by the Eurobioplex assay. The discrepancy for the genotype 5 sample was not typical of the genotype, as other genotype 5 specimens showed minimal difference (see the Bland–Altman plot of differences, Supplementary Figure 1).

**Table 2:** Side-by-side comparison of the in-house one-step and the EuroBioPlex HDV qRT-PCR assays with a panel of 15 specimens.

		Log_10_ IU/mL	
Panel sample	Sample HDV genotype	EuroBioPlex HDV qRT-PCR	Laboratory developed one-step HDV qRT-PCR
1	N/A	UND	UND
2	1	1.96	2.55
3	1	5.02	4.76
4	N/A	UND	UND
5	5	8.81	8.27
6	1	UND	1.66
7	N/A	UND	UND
8	5	0.55	2.58
9	1	7.25	7.12
10	1	3.61	3.59
11	N/A	UND	UND
12	1	5.79	4.83
13	5	5.14	5.39
14	N/A	UND	UND
Log_10_ 2.76 IU/mL WHO HDV standard	1	1.84	2.55

N/A, not applicable; UND, RNA undetectable.

Testing of the 2022 INSTAND quality assurance panel, consisting of 4 HDV specimens, also showed highly similar results by the one-step assay in comparison to the target value for each specimen (Table 3).

**Table 3:** Results of the 2022 INSTAND external quality assurance panel for HDV RNA detection using the laboratory developed one-step qRT-PCR assay.

INSTAND panel sample ID	Log_10_ IU/mL	
	INSTAND target (range)	One-step qRT-PCR
400,057	0.00 (0.0–0.0)	UND
400,058	3.61 (2.61–3.61)	3.28
400,059	3.92 (2.92–3.92)	3.67
400,060	4.19 (3.19–4.19)	3.99

## 4 Discussion

The method developed for quantification of HDV RNA in plasma or serum specimens uses a convenient one-step method of reverse transcription-PCR amplification resulting in highly sensitive, specific and accurate measurement of HDV RNA. Specifically, the developed method allows for quantification in IU/mL, which is needed for “standardization, harmonization and quality control of HDV RNA assays and patient management” (Paul-Ehrlich-Institut Regulation WHO Reference Material, 2024). The WHO international standard for HDV RNA is meant to be used as a calibrator for secondary reference materials (Paul-Ehrlich-Institut Regulation WHO Reference Material, 2024). This assay used the WHO standard to create a simplified conversion factor from copies/μL to IU/mL and included a quality control process to ensure synthetic HDV RNA calibrators and WHO RNA standards met criteria in each assay run. The use of one-step reverse transcription and PCR increased the ease of use of the assay and the time to result. One-step procedures have also been shown to increase assay precision compared to two-step methods (Le Gal et al., 2016). Most importantly, highly sensitive HDV RNA assays are necessary to meet an ideal clinical trial endpoint of undetectable HDV RNA (Umukoro et al., 2023).

Several recent reviews have described commercially available and LDT HDV RNA quantification assays that have been reported in the literature (Wedemeyer et al., 2023; Umukoro et al., 2023; Stelzl et al., 2021). The majority of LDTs measure HDV RNA in copies/mL and both LDT and commercial assays often utilize cloned DNA as an internal standard, which precludes the reverse transcription step and so has been shown to under-quantify HDV RNA (Maria et al., 2020). Currently there is no fully automated system available for HDV viral load measurement, which includes both extraction and amplification steps. However, LDT systems using the utility channel of the cobas automated platform (Roche Diagnostics) have been described, showing improved performance characteristics (Shang et al., 2012; Pflüger et al., 2021). Probit analysis and a log response curve was applied to the developed one-step qRT-PCR method to determine the LLoD to allow accurate reporting of HDV RNA results. Analytical characteristics of an LDT such as LLoD and LLoQ are critical to understand performance competency and limitations (Wedemeyer et al., 2023).

The decision to utilize a 2-point (2.38 and 7.38 log_10_ copies/μL) RNA calibration curve and a set conversion factor (16.5) to calculate IU/mL from copies/μL based on the calibration curve was made in order to, (1) increase the dynamic upper range of the assay, as the international standard value is limited to 5.76 log_10_ IU/mL, (2) reduce run-to-run variation in PCR slope efficiency observed with a standard curve composed of multiple RNA dilutions, and (3) implement quality control tracking of both calibrators and WHO HDV RNA standard controls to determine per run fitness and accuracy based on historical data. Quality control limits within each assay run were developed based on a weighted average and the square root of total variance of each calibrator following one-way ANOVA analysis of observations over a series of years. This method of analysis, suggested by Dimech et al. (2015), Dimech et al. (2018), Dimech (2021) for quality control of infectious disease testing, primarily serological testing, addresses the limitations of clinical chemistry-based statistical control limits for infectious disease assays involving multiple reagent lots (Dimech et al., 2018). As a fresh lot of synthesized target RNA calibration standards is periodically required, the potential variation introduced with each new lot is better controlled following result tracking and quality control analysis. Inclusion of the 3 WHO standard dilutions with quality control tracking ensures the slope and intercept parameters used to determine the conversion factor for copies/μL to IU/mL remain stable within each run.

Samples selected from archived clinical specimens having sequence data allowing the determination of HDV genotype were quantified using the one-step assay. Study samples of all 8 genotypes had quantifiable RNA, which was expected, as sufficient RNA had previously been amplified and sequenced. However, one genotype 6 sample was not amplified, possibly due to degradation following extended cold storage. Due to the very high nucleotide diversity of the HDV genome (Le Gal et al., 2017a) it has been shown that HDV molecular detection is challenging, with validated primer design a crucial element of assay development (Brichler et al., 2014). As the clinical specimens received by the National Microbiology Laboratory for HDV testing include a wide variety of genotypes (Osiowy et al., 2022), the developed quantitative assay utilized primer sequences that were largely conserved among all genotypes to control for genetically diverse HDV specimens. Comparison of HDV RNA viral load assays has shown drastic under-quantification of HDV genotypes, particularly genotype 1 subgenotypes found in Africa as well as genotypes 5 to 8 (Le Gal et al., 2016; Brichler et al., 2014; Brichler et al., 2013). A comparison of IU/mL values between the developed method and a commercial assay (Eurobioplex HDV qRT-PCR) was performed with specimens of HDV genotypes 1 and 5. It was observed that several samples undetectable or having <2 log_10_ IU/mL by Eurobioplex had consistently higher viral load values (>0.5 log_10_ IU/mL) by the one-step assay, while samples with viral load >3 log_10_ IU/mL often had slightly higher quantitative values with Eurobioplex, although not consistently, regardless of genotype. The Eurobioplex assay has previously been shown to report lower viral load values for genotypes 5 to 8, in comparison to the French National Reference Laboratory (Le Gal et al., 2017b). Future comparisons of the developed one step assay with commercially available HDV RNA quantitative assays should include a more extensive panel of HDV specimens and genotypes to further explore compatibilities.

HDV RNA quantification is used solely for patient management and not diagnosis as viral load measurement is normally only performed on patients having known chronic HDV infection. However, the ease of use and excellent sensitivity of the assay lends itself to being included in reflex testing following an HDV antibody positive result. Recently, there have been recommendations that all HBsAg-positive persons should be automatically screened for HDV antibodies at least once in their lifetime (European Association for the Study of the Liver, 2023; Razavi et al., 2023). However, as HDV RNA positivity has been confirmed to be associated with an increased risk of disease progression (Gish et al., 2024; Osiowy et al., 2022; Wranke et al., 2024), further reflex testing of HDV antibody positive persons for HDV RNA is necessary to fully assess newly diagnosed CHD patients. Such universal laboratory-based double reflex testing (i.e., reflex test first-time HBsAg-positive patients for HDV antibody followed by reflex test of all antibody positive samples for HDV RNA) for HDV has been recommended by international stakeholders [Coalition for Global Hepatitis Elimination;^2^ CDA Foundation Polaris Observatory^3^] and found to be the most practical and effective means to increase the diagnostic yield for HDV infection, particularly for Canada, which has a relatively low prevalence of HBsAg-positive individuals (Polaris Observatory Collaborators, 2024; Razavi et al., 2023).

Now that various new antiviral treatments for CHD are available on compassionate usage or through clinical trials, quantitative measurement of HDV RNA is critical for longitudinal analyses to understand patient response and therapeutic efficacy. However, although viral load may be measured in IU/mL, it remains necessary that patients are followed using the same viral load assay to have consistency in measurement over time with treatment, until commercial, standardized quantification methods are available.

There were several limitations that should be noted for this study and the developed method. The automated NucliSENS easyMag was implemented to support medium to high throughput RNA extraction for the demands of a reference laboratory, and so different methods of extraction were not explored. Thus, a manual extraction method may increase the sensitivity of detection, as has been observed previously for HDV RNA measurement (Anolli et al., 2024). Furthermore, the use of transcribed target RNA for internal assay calibration and quantification may not accurately represent HDV RNA secondary structure or the nature of the genomic RNA extracted from clinical samples (Wedemeyer et al., 2023). The synthetic RNA calibrators and the WHO HDV RNA standard, used to calculate the assay conversion factor, are HDV genotype 1, which may bias measurement, depending on the clinical isolate genotype. Although the developed assay was shown to detect and quantify clinical and synthetic specimens of all 8 HDV genotypes, the ability of the assay to accurately quantify all HDV subgenotypes was not explored. However, the HDV-infected population of Canada includes a variety of HDV genotype 1 (1a-1d), 2 (2a, 2b), and 5 (5a, 5b) subgenotypes (Osiowy et al., 2022), which would have been represented within the genotype panel investigated in this study. Furthermore, the use of extracted RNA from other viruses (HIV, HCV, etc.) was not included during development to evaluate viral specificity, nor was an amplification internal control included in the development of this assay; however, the use of the QuantiFast Pathogen kit (Qiagen) is an option for introduction of an internal control RNA during the qRT-PCR reaction (Scholtes et al., 2012).

In conclusion, the one-step qRT-PCR method developed for quantification of HDV RNA as IU/mL is a robust method providing sensitive and specific detection and quantification of all HDV genotypes. The method can easily be incorporated into double reflex testing, further allowing for HDV RNA quantification of newly diagnosed CHD patients for timely linkage to care and treatment. This type of testing design is desirable to address the underrecognized prevalence and burden of HDV and to work toward meeting the WHO 2030 goals for viral hepatitis elimination (World Health Organization, 2016).

## Data Availability

The datasets presented in this study can be found in online repositories. The names of the repository/repositories and accession number(s) can be found in the article/Supplementary material.

## Footnotes

^1^ https://nebiocalculator.neb.com/#!/ssrnaamt

^2^ www.globalhep.org

^3^ www.cdafound.org/polaris

## Supplementary material

SUPPLEMENTARY FIGURE 1 Bland-Altman plot of differences in HDV RNA log10 IU/mL results for a panel of specimens quantified by the Eurobioplex HDV qRT-PCR assay and the LDT one-step qRT-PCR assay. The mean difference between the two methods (bias) was calculated from the 10 panel samples having data and is shown on the Y axis. The 95% limits of agreement (95%CI -2.18 – 1.51) are shown as dotted lines. The mean bias was calculated to be -0.333. The average log10 IU/mL between the two methods for each panel sample is shown on the X axis. Red dots indicate HDV genotype 5 specimens among the panel, with panel sample #8 having a difference of -2.03. See Table 2 for detailed results used to create the Bland-Altman plot.
