# Supplementary material for: Laboratory development of an RNA quantitative RT-PCR assay reporting in international units for hepatitis D virus
Source: Front Microbiol. 2024 Nov 20;15:1472826. doi: 10.3389/fmicb.2024.1472826 (PMC11615724; doi:10.3389/fmicb.2024.1472826)
Supplement: Supplementary file 1 [file Table_1.docx]

| **HDV Genotype** | **Specimen ID Code** | **Log_10_ IU/mL** |
| --- | --- | --- |
| HDV Genotype 1 | H16/3517 | 3.55 |
|  | H16/3147 | 6.27 |
|  | H14/4292 | 5.18 |
|  | H16/3723 | 1.01* |
|  | H16/3046 | 1.62 |
| HDV Genotype 2 | H3752/09 | 2.79 |
|  | H15/3171 | 3.75 |
|  | H15/2382 | 1.21 |
|  | H16/3260 | 2.29 |
|  | H16/2421 | 3.92 |
|  | H15/4590 | 3.91 |
| HDV Genotype 3 | gBlock 0.01 pg/µL | 6.36 |
|  | gBlock 0.01 pg/µL | 6.36 |
|  | gBlock 0.001 pg/µL | 5.44 |
|  | gBlock 0.001 pg/µL | 5.46 |
| HDV Genotype 4 | gBlock 0.01 pg/µL | 6.58 |
|  | gBlock 0.01 pg/µL | 6.62 |
|  | gBlock 0.001 pg/µL | 5.72 |
|  | gBlock 0.001 pg/µL | 5.71 |
| HDV Genotype 5 | H16/3322 | 6.45 |
|  | H13/5946 | 5.07 |
|  | H14/4849 | 2.26 |
| HDV Genotype 6 | H12/2952 | UND |
|  | H15/6123 | 6.09 |
|  | H16/1927 | 5.39 |
| HDV Genotype 7 | H14/6361 | 3.90 |
|  | H16/1922 | 4.90 |
|  | H13/4898 | 2.84 |
|  | H16/0899 | 5.19 |
| HDV Genotype 8 | H3943/11 | 3.61 |

**Supplementary Table 1.** Detection and quantification of HDV RNA in clinical specimens or synthetic linear dsDNA (gBlock) of all 8 HDV genotypes by the developed one-step qRT-PCR assay.

*Value is below the calculated LLoD; UND: undetectable
