# Supplementary material for: Laboratory development of an RNA quantitative RT-PCR assay reporting in international units for hepatitis D virus
Source: Front Microbiol. 2024 Nov 20;15:1472826. doi: 10.3389/fmicb.2024.1472826 (PMC11615724; doi:10.3389/fmicb.2024.1472826)
Supplement: Supplementary file 2 [file Table_2.docx]

**Supplementary Table 2.** Determination of the conversion factor from copies/µL to IU/mL, following calculation of IU/mL of synthetic RNA dilutions tested by the one-step qRT-PCR assay.

| **Synthetic HDV**  **RNA Log_10_ copies/µL** | **Synthetic HDV**  **RNA copies/µL** | **Calculated**^1^  **Log_10_ IU/mL** | **Calculated**  **IU/mL** | **IU/mL**^2^  **Copies/µL** |
| --- | --- | --- | --- | --- |
| 1.375  2.375  3.375  4.375  5.375  6.375  7.375  8.375 | 2.37E+01  2.37E+02  2.37E+03  2.37E+04  2.37E+05  2.37E+06  2.37E+07  2.37E+08 | 2.673  3.600  4.610  5.569  6.592  7.601  8.528  9.555 | 4.71E+02  3.98E+03  4.07E+04  3.71E+05  3.91E+06  3.99E+07  3.37E+08  3.59E+09 | 19.89  16.78  17.18  15.64  16.48  16.83  14.22  15.14 |
| ^1^Calculated from the mean Ct of each synthetic RNA dilution (over approximately 20 determinations per dilution) and the slope (-3.46) and intercept (40.934) of the line created from the mean Ct of WHO HDV RNA standards (1.76, 2.76, 4.76 log10 IU/mL; approximately 20 determinations per dilution). ^2^Mean IU/mL ÷ copies/µL = 16.52 | | | | |
