# Supplementary figures and images for: Laboratory development of an RNA quantitative RT-PCR assay reporting in international units for hepatitis D virus
Source: Front Microbiol. 2024 Nov 20;15:1472826. doi: 10.3389/fmicb.2024.1472826 (PMC11615724; doi:10.3389/fmicb.2024.1472826)

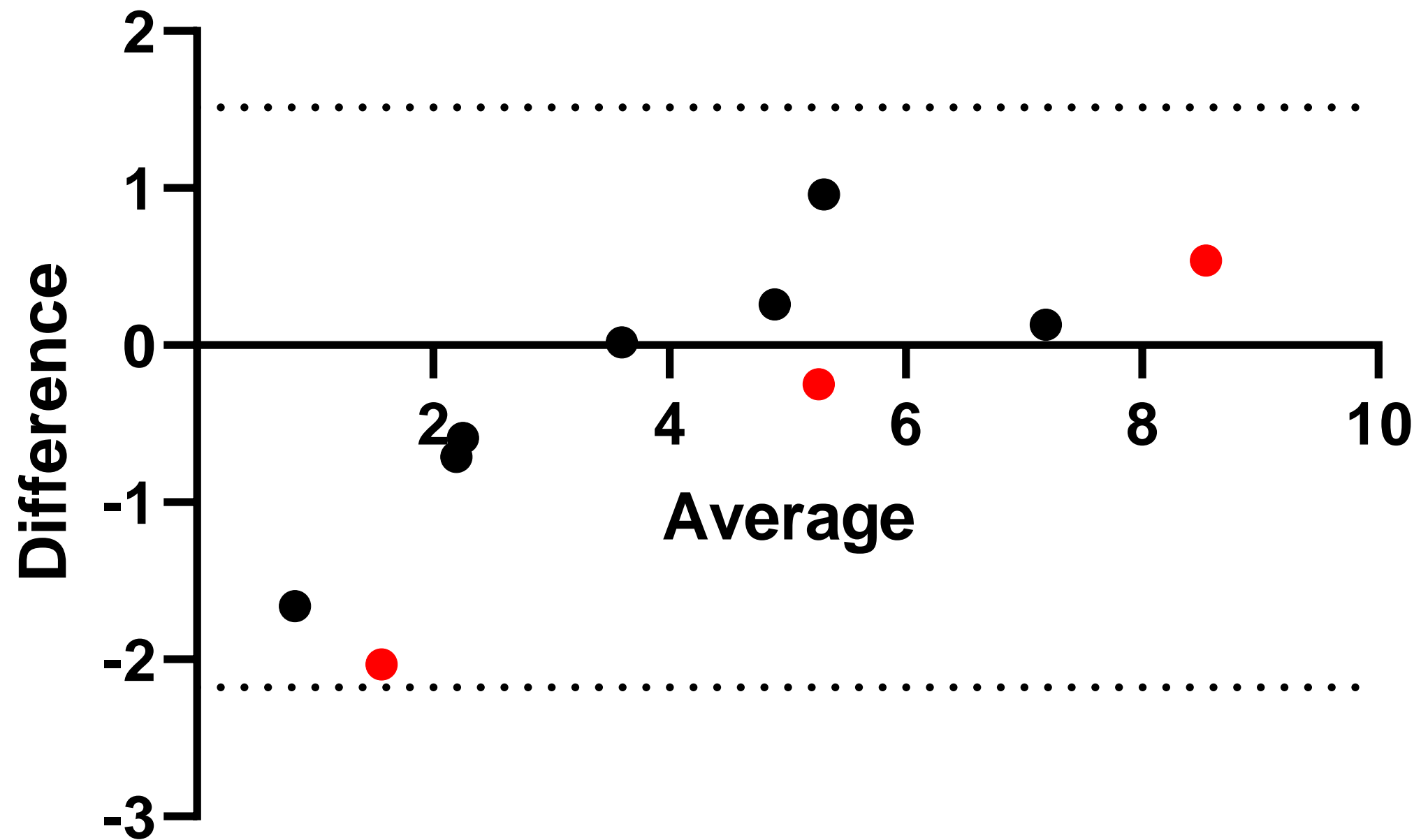

Supplement: SUPPLEMENTARY FIGURE 1 — Bland-Altman plot of differences in HDV RNA log10 IU/mL results for a panel of specimens quantified by the Eurobioplex HDV qRT-PCR assay and the LDT one-step qRT-PCR assay. The mean difference between the two methods (bias) was calculated from the 10 panel samples having data and is shown on the Y axis. The 95% limits of agreement (95%CI -2.18 – 1.51) are shown as dotted lines. The mean bias was calculated to be -0.333. The average log10 IU/mL between the two methods for each panel sample is shown on the X axis. Red dots indicate HDV genotype 5 specimens among the panel, with panel sample #8 having a difference of -2.03. See Table 2 for detailed results used to create the Bland-Altman plot. [file Image_1.pdf]
